# Analysis of *ESR1* and *PIK3CA* mutations in plasma cell-free DNA from ER-positive breast cancer patients

**DOI:** 10.18632/oncotarget.18479

**Published:** 2017-06-14

**Authors:** Takashi Takeshita, Yutaka Yamamoto, Mutsuko Yamamoto-Ibusuki, Mai Tomiguchi, Aiko Sueta, Keiichi Murakami, Yoko Omoto, Hirotaka Iwase

**Affiliations:** ^1^ Department of Breast and Endocrine Surgery, Graduate School of Medical Science, Kumamoto University, Honjo, Chuo-Ku, Kumamoto, Japan; ^2^ Department of Molecular-Targeting Therapy for Breast Cancer, Kumamoto University Hospital, Honjo, Chuo-Ku, Kumamoto, Japan; ^3^ Department of Endocrinological and Breast Surgery, Graduate School of Medical Science, Kyoto Prefectural University of Medicine, Hirokoji Agaru, Kawaramachi-Dori, Kamigyo-Ku, Kyoto, Japan

**Keywords:** estrogen receptor-positive metastatic breast cancer, acquired endocrine therapy resistance, cell-free DNA, ESR1 mutations, PIK3CA mutations

## Abstract

**Background:**

The measurement of *ESR1* and *PIK3CA* mutations in plasma cell-free DNA (cfDNA) has been studied as a non-invasive method to quickly assess and monitor endocrine therapy (ET) resistant metastatic breast cancer (MBC) patients.

**Methods:**

The subjects of this retrospective study were a total of 185 plasma samples from 86 estrogen receptor-positive BC patients, of which 151 plasma samples were from 69 MBC patients and 34 plasma samples were from 17 primary BC (PBC) patients. We developed multiplex droplet digital PCR assays to verify the clinical significance of *ESR1* and *PIK3CA* mutations both in a snapshot and serially in these patients.

**Results:**

cfDNA *ESR1* and *PIK3CA* mutations were found in 28.9% and 24.6 % of MBC patients, respectively. The relation between *ESR1* or *PIK3CA* mutations and clinical features showed that *ESR1* mutations occurred mostly in patients previously treated by ET, which was not the case for *PIK3CA* mutations. The analysis of the clinical impact of those mutations on subsequent lines of treatment for the 69 MBC patients revealed that both *ESR1* and *PIK3CA* mutations detection were related to a shorter duration of ET effectiveness in univariate analysis but only for *ESR1* mutations in multivariate analysis. The monitoring of cfDNA in a subset of 52 patients showed that loss of *ESR1* mutations was related to a longer duration of response, which was not the case for *PIK3CA* mutations.

**Conclusions:**

We have demonstrated the clinical significance of on-treatment *ESR1* mutations both in a snapshot and serially in comparison with *PIK3CA* mutations.

## INTRODUCTION

Approximately 80% of breast cancers (BCs) express the estrogen receptor alpha (ERα), encoded by the *ESR1* gene, and endocrine therapy (ET) with selective ER modulators (SERMs) or aromatase inhibitors (AIs) is the mainstay of treatment for this group of patients because of their effectiveness balanced against their side effects. However, ET resistance occasionally occurs during the treatment of early BC and inevitably results in metastatic BC (MBC) [1]. *ESR1* ligand binding domain (LBD) mutations constitutively activate the ER in a ligand-independent fashion [2–4] and they have attracted attention as a mechanism of ET resistance in MBC. These mutations were originally reported almost two decades ago [5–8], and recent large-scale next-generation sequencing (NGS) revealed that *ESR1* mutations are present in approximately 20-50% of metastatic tissue samples treated with endocrine agents while these variants are absent or only present at very low frequencies in primary tumor samples [2–4]. These features indicate that the presence of *ESR1* mutations should be assessed in metastatic lesions. Circulating cell-free DNA (cfDNA) has been proposed to carry a comprehensive picture of metastatic tumor cells and genomic analysis of plasma cfDNA has been realized as a non-invasive method to quickly assess the mutational profiles and monitor molecular changes under treatment, using recent developments in digital genomic technologies [9]. Therefore, if *ESR1* mutation status in cfDNA is predictive of response to ET, monitoring of this marker could be a useful method of informing treatment plans for subsequent metastatic disease.

*PIK3CA* is an oncogene that encodes the p110α component of phosphatidylinositol 3-kinase (PI3K) and *PIK3CA* is a representative frequently-mutated gene, whose frequencies are 20% to 40% of all BCs [10, 11]. Recently, in phase III randomized trials, the clinical significance of *ESR1* mutations have been reported in the comparison with *PIK3CA* mutations. In alteration frequency in metastatic versus primary tumors in the BOLERO-2 cohort, Hortobagyi et al. demonstrated that *PIK3CA* mutations had the highest frequency in PBCs and MBCs and that *ESR1* mutations had higher frequency in MBCs than in PBCs [12]. More recently, in the BOLERO-2 study, Chandarlapaty and colleagues found that 28.8% (155/541) of ER-positive MBC patients had *ESR1* mutations in plasma cfDNA [13] and 43.3% (238/550) of ER-positive MBC patients had *PIK3CA* mutations in plasma cfDNA [14]. They also demonstrated that the difference of clinical features between *ESR1* and *PIK3CA* mutations, namely, progression free survival (PFS) benefit of mammalian target of rapamycin (mTOR) inhibitor everolimus was maintained irrespective of *PIK3CA* mutations, but that was decreased according to the presence of *ESR1* mutations [13, 14]. In another two phase III randomized trials, Fribbens and colleagues assessed *ESR1* mutations in cfDNA using digital PCR (dPCR) [15]. *ESR1* mutations were found in the plasma of 39.1% of patients (63/161) in the SoFEA study and 25.3% (91/360) in the PALOMA3 study. *PIK3CA* mutations were found in the plasma of 33% (129/395) of patients in the PALOMA3 study [16]. They also reported the effectiveness of the target drug by having the mutations or not. In the SoFEA study, patients with *ESR1* mutations had improved PFS after taking fulvestrant compared with exemestane. In the PALOMA3 study, fulvestrant plus the CDK4/6-inhibitor palbociclib improved PFS regardless of the genomic status of *ESR1* or *PIK3CA* [15, 16].

In this retrospective study, we demonstrated the clinical significance of on-treatment hotspot *ESR1* LBD mutations both in a snapshot and serially in 185 plasma samples from 86 patients in comparison with the hotspot mutation status of *PIK3CA* using multiplex droplet dPCR (ddPCR) assays. To our knowledge, this is the leading comparative study to identify the clinical significance of multiplex ddPCR detection of *ESR1* mutations and *PIK3CA* mutations in plasma samples.

## RESULTS

### *ESR1* mutations in cfDNA baseline plasma samples

**Figure 1:**
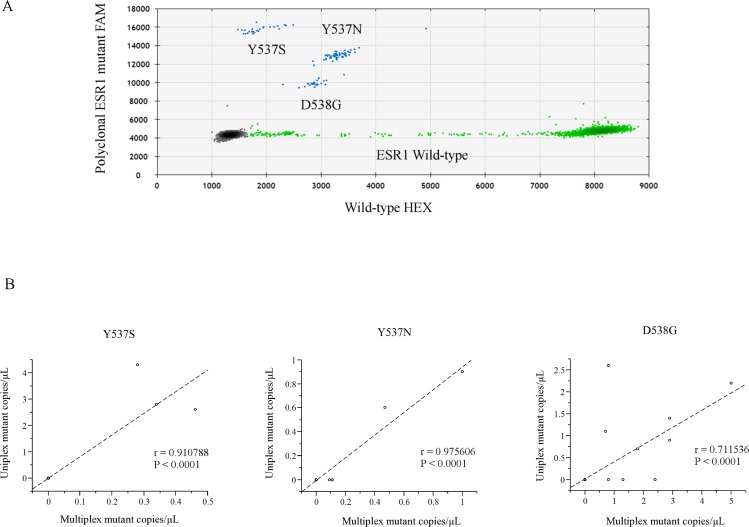
**A.** Representative ddPCR analysis of polyclonal *ESR1* mutations. The presence of all three hotspot LBD *ESR1* mutations (Y537S, Y537N, and D538G) was confirmed by uniplex assays (Supplementary Figure S1). In each plot, green dots represent HEX-labeled wild type DNA, blue dots represent FAM-labeled mutant DNA, and black dots are droplets with no DNA incorporated. **B**. Comparison of each LBD *ESR1* mutation between uniplex and multiplex ddPCR assays from a validation subset of 26 women (62 blood samples). Abbreviations; ddPCR, droplet digital polymerase chain reaction; LBD, ligand binding domain; cfDNA.

We developed a sensitive and quantitative multiplex ddPCR assay to screen for 3 hotspot mutations in the LBD of *ESR1*. Figure 1A and Supplementary Figure S1 show the comparative analysis of a dilution series of each indicated synthetic *ESR1* mutation oligonucleotide by ddPCR. We used serial dilutions of three hotspot *ESR1* LBD mutant recombinant DNAs: *ESR1* Y537S, Y537N, and D538G, and analyzed them using a multiplex *ESR1* mutant detection probe, which could simultaneously detect *ESR1* Y537S, Y537N, and D538G, confirming that this assay was able to detect as few as three copies of the mutant allele in an abundance of wild-type DNA. There was no cross-reactivity for the detection of each *ESR1* mutations.

Next, to validate the utility of the multiplex ddPCR assay, a subset of 26 women (62 blood samples), who were previously evaluated using a uniplex *ESR1* mutant detection probe [17], were analyzed using a multiplex *ESR1* mutant detection probe, and a statistically significant correlation between uniplex and multiplex ddPCR assays was found (Figure 1B, Supplementary Table S1: Y537S, κ = 1.0, r = 0.91, *P* < 0.0001; Y537N, κ = 1.0, r = 0.55, *P* < 0.0001; D538G, κ = 0.77, r = 0.71, *P* < 0.0001)

### Detection of *ESR1* mutations and *PIK3CA* mutations in cfDNA of women with ER-positive BC

**Figure 2:**
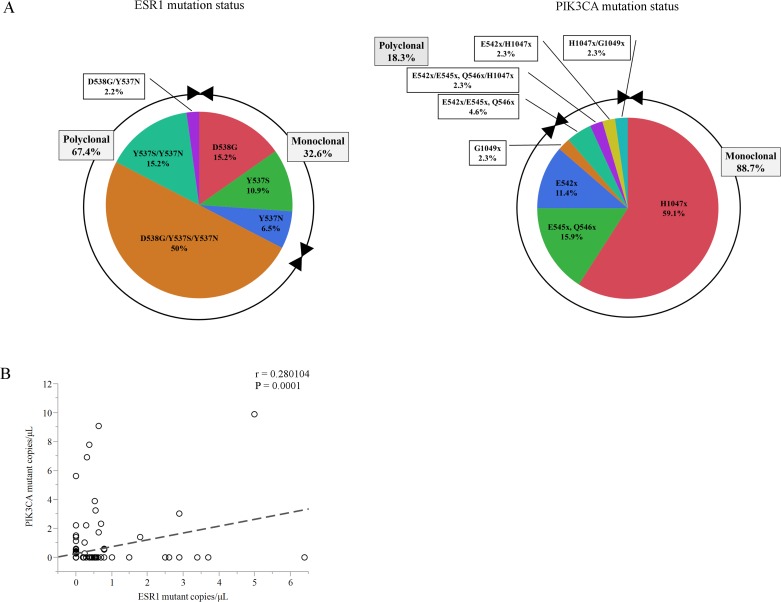
**A.** Chart showing the percentage of *ESR1* mutations and *PIK3CA* mutations in plasma cfDNA in this cohort. **B**. Comparison of the number of copies/μL between total *ESR1* mutations and total *PIK3CA* mutations in plasma from a subset of 86 women (185 blood samples). Abbreviations; cfDNA, cell-free DNA; H1047x, H1047L/R/Y; E545x Q546x, E545V/G/A/Q/K Q546L/R/P/E/K; E542x, E542K/V; G1049x, G1049R/S.

A total of 86 patients (185 plasma samples) with breast carcinoma, who had the ECOG scale of Performance status 0 or 1, were enrolled in this study. Participants comprised 17 women (34 plasma samples) with primary BC (PBC) and 69 women (151 plasma samples) with MBC. The patient demographics and baseline characteristics of PBC and MBC are presented in Table 1. The median age of the patients at first blood draw was 67 years (range, 41-82) in the PBC group and 59 years (range, 32-85) in the MBC group. Of the clinical stage at diagnosis 69 MBC patients, 20 patients (30 %) were categorized as stage IV. All 17 PBC patients were treated by neoadjuvant ET. Of the MBC patients, 79.7 % (55/69) were previously treated with AIs, 60.9% (42/69) were previously treated with SERMs, and 56.5% (39/69) were previously treated with both AIs and SERMs, but 23.2 % (16/69) had not previously received any ET. The median duration of follow-up was 33 months (range, 13-101 months) in the PBC group and 49 months (range, 11-268 months) in the MBC group. There was no recurrence during the observation period in any of the PBC patients. Concerning the multiplex assay, it should be tested using negative control to help defining the background noise [18]. Therefore, we verified the multiplex ddPCR assay using PBC patients whose allele frequency (AF) of *ESR1* mutations is very low [2–4]. We found 4 *PIK3CA* mutations in cfDNA, but we did not find any *ESR1* mutations in the PBC group (Supplementary Table S3). Figure 2 shows the percentage of *ESR1* mutations and *PIK3CA* mutations in plasma cfDNA of BC subsets. *ESR1* mutations and *PIK3CA* mutations were evaluated using multiplex mutant detection probes. *ESR1* mutations in cfDNA were detected in 30.5 % (46/185) of all samples (Supplementary Table S2)*. ESR1* Y537S, Y537N, and D538G were found in 76.1%, 73.9%, and 67.4% of samples with *ESR1* mutations, respectively (Figure 2A). Among patients with any detectable *ESR1* mutations, 67.4 % (31/46) had two or more mutations (17.4% (8/46) had two mutations and 50% (23/46) had more than three mutations). *PIK3CA* mutations in cfDNA were detected in 23.8 % (44/185) of all samples (Supplementary Table S2). *PIK3CA* H1047L/R/Y, E545V/G/A/Q/K Q546L/R/P/E/K, E542K/V, and G1049R/S were found in 65.9%, 22.7%, 20.5% and 6.8% of *PIK3CA* mutant samples, respectively. The majority of cfDNA samples (88.7 %, 39/44) had only a single-site detectable *PIK3CA* mutation in cfDNA and exhibited markedly less heterogeneity than *ESR1* mutations (Figure 2A, Supplementary Table S2). Nine patients had co-mutation (*ESR1* and *PIK3CA* mutations) over the all of treatment. However, there was no statistically significant correlation of copies/μL between *ESR1* mutations and *PIK3CA* mutations in plasma cfDNA (r = 0.28, *P* = 0.0001) (Figure 2B, Supplementary Table S2).

**Table 1:** Patient characteristics

	No. of patients (%)		
**Variables**	Total	PBC	MBC
	(N = 86)	(N = 17)	(N = 69)
**Age at biopsy**			
Median (range)	58 (31–82)	67 (41–82)	59 (32–85)
**Clinical Stage at diagnosis**			
I	16 (18.6)	4 (23.5)	12 (17.4)
II	36 (41.8)	11 (64.7)	25 (36.2)
III	14 (16.3)	2 (11.8)	12 (17.4)
IV	20 (23.3)	0	20 (30)
**Histological type**			
Invasive ductal	79 (91.9)	13 (76.5)	66 (95.5)
Invasive lobular	2 (2.3)	1 (5.9)	1 (1.5)
Mucinous	4 (4.7)	3 (17.6)	1 (1.5)
Neuroendocrine therapy	1 (1.1)	0	1 (1.5)
**Histological grade**			
1	27 (31.4)	5 (29.4)	22 (31.9)
2	37 (43.0)	11 (64.7)	26 (37.7)
3	16 (18.6)	0	16 (23.2)
Lobular	2 (2.3)	1 (5.9)	1 (1.4)
Unknown	4 (4.7)	0	4 (5.8)
**Percentage of ERα median (25%, 75%)**	86	90 (90–95)	90 (65–95)
**Percentage of PgR median (25%, 75%)**	86	50 (7.5–80)	30 (0–70)
**HER2**			
Negative	76 (88.4)	16 (94.1)	60 (87)
Positive	10 (11.6)	1 (5.9)	9 (13)
**Visceral involvement**			
No	25 (29.1)	17 (100)	18 (26.1)
Yes	51 (70.9)	0	51 (73.9)
**Bone involvement**			
No	48 (55.8)	17 (100)	31 (44.9)
Yes	38 (44.2)	0	38 (55.1)
**Number of metastatic lesions**			
0	17 (19.8)	17 (100)	0
1	8 (9.3)	0	8 (11.6)
2	25 (29.1)	0	25 (36.2)
3 ≤	36 (41.8)	0	36 (52.2)
**Prior endocrine therapy**			
SERM	42 (48.8)	0	42 (60.9)
AI	72 (83.7)	17 (100)	55 (79.7)
Both AI and SERM	39 (45.3)	0	39 (56.5)
**Number of prior endocrine regimens**			
0	16 (18.6)	0	16 (23.2)
1	29 (33.7)	17 (100)	12 (17.4)
2	5 (5.8)	0	5 (7.3)
3 ≤	36 (41.9)	0	36 (52.1)
**Number of prior courses of chemotherapy**			
0	52 (60.5)	17 (100)	35 (50.7)
1	16 (18.6)	0	16 (23.2)
2	3 (3.5)	0	3 (4.4)
3 ≤	15 (17.4)	0	15 (21.7)

Abbreviations: PBC, primary breast cancer; MBC, metastatic breast cancer; ERα, estrogen receptor alpha; PgR, progesterone receptor; HER2, human epidermal growth factor receptor 2; SERM, selective estrogen receptor modulator; AI, aromatase inhibitor.

### Association of *ESR1* and *PIK3CA* mutations with clinical features

Table 2 shows *ESR1* and *PIK3CA* mutations as they relate to baseline clinical and pathological features. In the snapshot study, we analyzed the latest genetic state in cfDNA in each BC patient. Plasma *ESR1* mutations were found in 28.9% (20/69) of MBC patients while plasma *PIK3CA* mutations were found in 24.6 % (17/69). The presence of plasma *ESR1* mutations was associated with several clinicopathological parameters. Ninety-five percent of patients with *ESR1* mutations had visceral disease, whereas only 65.4 % of *ESR1* wild-type (WT) patients had visceral disease (*P* = 0.011). In addition, all patients with *ESR1* mutations had resistance to prior AI therapy compared with 71.4% of *ESR1* WT patients (*P* = 0.0074); 85% of patients with *ESR1* mutations had resistance to prior SERM therapy compared with 51% of *ESR1* WT patients (*P* = 0.0087), and 80% of patients with *ESR1* mutations had been treated with a prior endocrine regimen three or more times compared with 40.3% of *ESR1* WT patients. Other demographic and clinical parameters were generally balanced between patients with *ESR1* mutations and *ESR1* WT patients, including age, histological grade, the expression of ER and progesterone receptor (PgR), human epidermal growth factor receptor 2 (HER2) positivity, bone involvement, the number of metastatic lesions, the number of subsequent endocrine regimens, and the number of prior and subsequent chemotherapies and total duration of chemotherapies. In similar analyses, *PIK3CA* mutations in cfDNA generally were not associated with particular demographic and clinical features, with the exception of visceral involvement (*P* = 0.029). In the PBC group, the presence of *PIK3CA* mutations in cfDNA was not associated with particular clinicopathological features (Supplementary Table S3).

**Table 2:** Patients and clinicopathological characteristics associated with *ESR1* and *PIK3CA* mutations in cfDNA of 69 MBC patients

	No. of patients (%)					
**Variables**	**ESR1**		***P*-value**	**PIK3CA**		***P*-value**
	**wild type**	**mutation**		**wild type**	**mutation**	
	(***N* = 49)**	(***N* = 20)**		(***N* = 52)**	(***N* = 17)**	
**Age at biopsy**						
Median (range)	59 (50.5–65)	60 (51.3–70.5)	0.30	58.5 (51-66)	61 (48.5-65.5)	0.92
**Histological grade**						
1	18 (38.3)	4 (23.5)	0.55	14 (26.9)	8 (47.1)	0.24
2	18 (38.3)	8 (47.1)		22 (42.3)	4 (23.5)	
3	11 (23.4)	5 (29.4)		12 (23.1)	4 (23.5)	
**Median percentage of ERα (25%, 75%)**	90 (70–95)	90 (52.5–100)	0.85	90 (60-95)	90 (80-95)	0.54
**Median percentage of PgR (25%, 75%)**	40 (0–70)	7.5 (0–70)	0.41	40 (0-70)	5 (0-75)	0.63
**HER2-positive**	6 (12.2)	4 (20)	0.41	8 (15.3)	2 (11.8)	0.71
**Visceral involvement**	32 (65.3)	19 (95)	0.011 a	35 (67.3)	16 (94.1)	0.029 a
**Bone involvement**	26 (53.1)	12 (60)	0.6	29 (55.8)	9 (52.9)	0.84
**Number of metastatic lesions**						
1	8 (16.3)	0	0.12	7 (13.5)	1 (5.9)	0.93
2	18 (36.7)	7 (35)		19 (36.5)	6 (36.2)	
3 ≤	23 (47)	13 (65)		26 (50)	10 (58.8)	
**Prior endocrine therapy**						
SERM	25 (51)	17 (85)	0.0087 a	29 (55.8)	13 (76.5)	0.12
AI	35 (71.4)	20 (100)	0.0074 a	40 (76.9)	15 (88.2)	0.31
Both AI and SERM	22 (44.9)	17 (85)	0.0014 a	27 (51.9)	12 (70.6)	0.17
**Number of prior endocrine regimens**						
0	16 (32.7)	0	0.041 a	15 (28.8)	1 (5.9)	0.052
1, 2	13 (26.5)	4 (20)		14 (26.9)	3 (17.6)	
3 ≤	20 (40.8)	16 (80)		23 (44.2)	13 (76.5)	
**Number of subsequent endocrine regimen**						
0	4 (8.2)	5 (25)	0.16	5 (28.8)	4 (23.5)	0.11
1, 2	22 (44.9)	8 (40)		21 (26.9)	9 (17.6)	
3 ≤	23 (46.9)	7 (35)		26 (44.2)	4 (76.5)	
**Number of prior courses of chemotherapy**						
0	25 (51)	10 (50)	0.95	30 (57.7)	5 (29.4)	0.13
1, 2	13 (26.5)	6 (30)		12 (23.1)	7 (41.2)	
3 ≤	11 (22.4)	4 (20)		10 (19.2)	5 (29.4)	
**Number of subsequent courses of chemotherapy**						
0	18 (36.7)	7 (35)	0.40	19 (36.5)	6 (35.3)	0.96
1, 2	21 (42.9)	6 (30)		21 (40.4)	6 (35.3)	
3 ≤	10 (20.4)	7 (35)		12 (23.1)	5 (29.4)	
**Duration of total chemotherapy (months)**						
Median (range)	13 (0-118)	12.5 (0-38)	0.90	13 (0-60)	14 (0-118)	0.69

Abbreviations: ERα, estrogen receptor alpha; PgR, progesterone receptor; HER2, human epidermal growth factor receptor 2; SERM, selective estrogen receptor modulator; AI, aromatase inhibitor.

a Factor showing statistical significance (*P* < 0.05).

### Association of *ESR1* and *PIK3CA* mutations with clinical outcome

**Figure 3:**
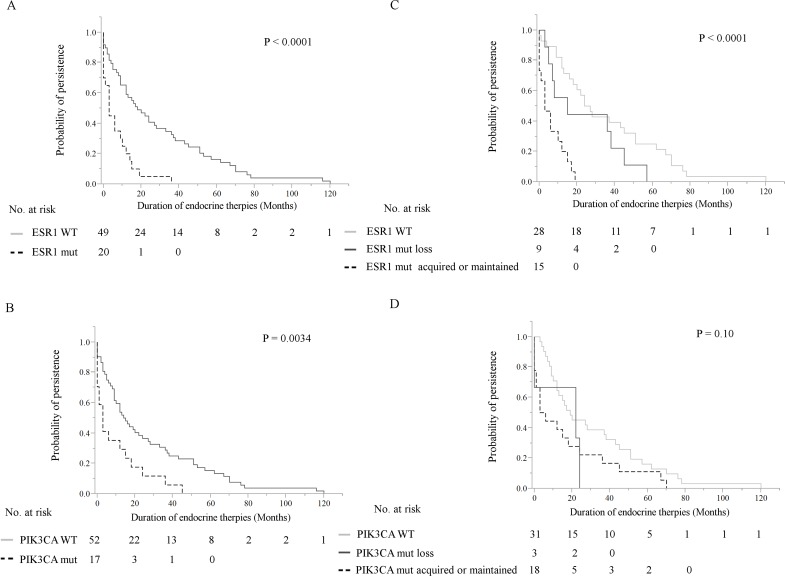
Kaplan-Meier plots of the association of *ESR1* mutations and *PIK3CA* mutations in cfDNA with duration of ET effectiveness in this cohort These were verified by the log-rank test. **A**, **B**. Patients with detectable plasma *ESR1* mutations (*P* < 0.0001) (A) and *PIK3CA* mutations (*P* = 0.0034) (B) showed significantly shorter duration of ET effectiveness in 69 MBC patients. **C**., **D**. Tracking analysis of cfDNA *ESR1* mutations and *PIK3CA* mutations in 52 breast cancer patients with longitudinal samples. Patients were grouped into those who did not have any *ESR1* or *PIK3CA* mutations over the course of treatment, those in whom *ESR1* or *PIK3CA* mutations were maintained or acquired, and those in whom *ESR1* or *PIK3CA* mutations disappeared in cfDNA. C. Patients in the loss of cfDNA *ESR1* mutations group had a longer duration of ET effectiveness than patients with acquired or maintained numbers of cfDNA *ESR1* mutations, but had a shorter duration of ET effectiveness than patients without mutations over the course of treatment (*P* < 0.0001). D. There was no statistically significant differences in these three groups; no *PIK3CA* mutations during treatment group, the loss of cfDNA *PIK3CA* mutations group, and the acquired or maintained numbers of cfDNA *PIK3CA* mutations group. Abbreviations: ET, endocrine therapy; cfDNA, cell-free DNA; MBC, metastatic breast cancer.

We retrospectively analyzed whether *ESR1* and *PIK3CA* mutations detected in cfDNA were associated with differential benefit in relation to the duration of ET effectiveness (Figure 3, Supplementary Figure S2). In that analysis, discontinuation of ETs caused by local recurrences, distant metastases, and disease progression at any site following the blood draw were considered as an event. These were tested by Kaplan-Meier analysis and verified by the log-rank test. Patients with detectable plasma *ESR1* mutations (*P* < 0.0001) and *PIK3CA* mutations (*P* = 0.0034) showed statistically significant shorter duration of ET effectiveness (Figure 3A, 3B). The Cox hazards model analysis of duration of ET effectiveness is shown in Table 3. The presence of *ESR1* mutations in cfDNA was a significant prognostic parameter in univariate analysis (hazard ratio (HR): 3.2, 95% confidence interval (CI): 1.76-5.71, *P* = 0.0002) and in multivariate analysis (HR: 2.04, 95% CI: 1.08-3.83, *P* = 0.029). The presence of *PIK3CA* mutations in cfDNA was a significant prognostic parameter in univariate analysis (HR: 2.25, 95% CI: 1.24-3.94, *P* = 0.0091), but showed only a marginal relationship in multivariate analysis (HR: 1.78, 95% CI: 0.96-3.18, *P* = 0.066). Other clinical parameters were not found to be statistically significant in univariate analysis, with the exception of prior SERM (HR: 2.30, 95% CI: 1.39-3.87, *P* = 0.0011), prior AI (HR: 2.02, 95% CI: 1.14-3.81, *P* = 0.015), and prior both AIs and SERMs (HR: 2.89, 95% CI: 1.73-4.92, *P* < 0.0001), nor in multivariate analysis. We also examined whether patients with a higher AF of *ESR1* mutations or *PIK3CA* mutations in cfDNA showed differential outcomes in duration of ET effectiveness. The box-plots for the AF of *ESR1* mutations and *PIK3CA* mutations are shown in Supplementary Figure S2. The median AF of *ESR1* mutations was 8.66 % (range, 0.35-78.5) and the median AF of *PIK3CA* mutations was 10.3% (range, 2.57-35), respectively. The dichotomized *ESR1* and *PIK3CA* AF (cutoff each AF > median) did not show a clear difference in duration of ET effectiveness.

**Table 3:** Univariate and multivariate analysis of factors associated with discontinuity in endocrine therapy in women with breast cancer

Variables			Univariate analysis			Multivariate analysis		
		**Value**	**HR**	**95%CI**	***P* value**	**HR**	**95%CI**	***P* value**
**Age at biopsy**	(ref = ≤ 50)	> 50	1.26	0.72-2.34	0.43			
**Histological grade**	(ref = 1,2)	3	0.96	0.52-1.69	0.91			
**ER (IHC)**	(ref = median)	≥ 90%	1.28	0.77-2.10	0.34			
**PgR (IHC)**	(ref = median)	≥ 30%	0.99	0.61-1.60	0.97			
**Visceral involvement**	(ref = No)	Yes	1.04	0.61-1.84	0.90			
**Bone involvement**	(ref = No)	Yes	1.22	0.76-1.99	0.41			
**Number of metastatic lesions**	(ref = 1, 2)	≥ 3	1.20	0.74-1.94	0.46			
**Prior endocrine therapy**								
**SERM**	(ref = No)	Yes	2.30	1.39-3.87	0.0011 ^a^	0.70	0.16-2.3	0.59
AI	(ref = No)	Yes	2.02	1.14-3.81	0.015 ^a^	1.0	0.46-2.3	0.99
Both AI and SERM	(ref = No)	Yes	2.89	1.73-4.92	< 0.0001 ^a^	2.96	0.75-15.1	0.13
***ESR1* genomic state**	(ref = WT)	Mut	3.2	1.76-5.71	0.0002 ^a^	2.04	1.08-3.83	0.029 ^a^
***ESR1* MAF (%)**	(ref = < Median)	Median >	0.73	0.30-1.82	0.51			
***PIK3CA* genomic state**	(ref = WT)	Mut	2.25	1.24-3.94	0.0091 ^a^	1.78	0.96-3.18	0.066
***PIK3CA* MAF (%)**	(ref = < Median)	Median >	0.76	0.29-2.04	0.58^a^			

(Cox proportional hazards model)

Abbreviations: HR, hazard ratio; 95%CI, 95% confidence interval; ER, estrogen receptor; IHC, immunohistochemistry; PgR, progesterone receptor; SERM, selective estrogen receptor modulator; AI, aromatase Inhibitor; MAF, mutant allele frequency; Mut, mutation.

^a^ Factor showing statistical significance.

### Tracking cfDNA *ESR1* mutations in 52 MBC patients with longitudinal samples

**Figure 4:**
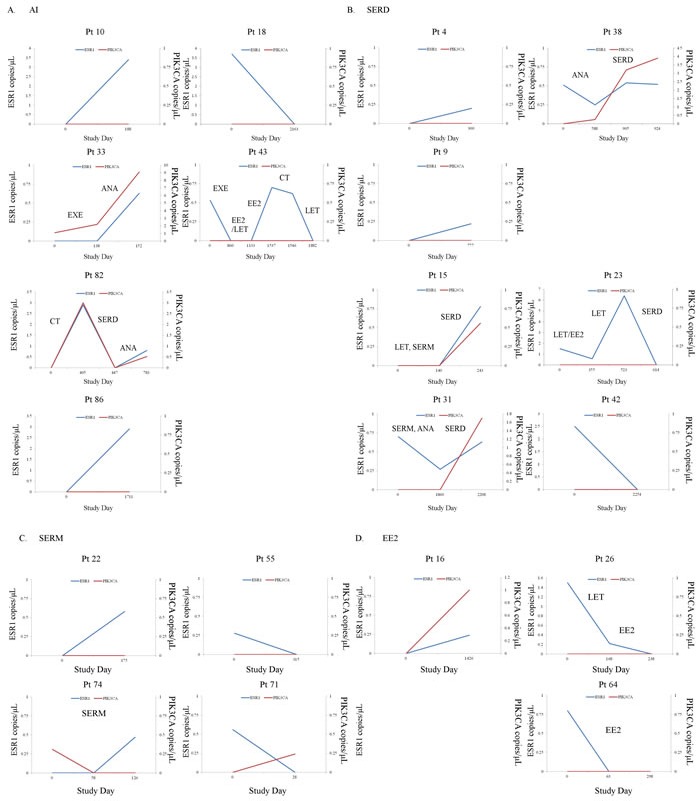
**A**.-**D**. Intrapatient changes of the numbers of copies/μL of *ESR1* and *PIK3CA* mutations under treatment with each representative therapeutic drug in 21 patients with longitudinal data. A. Patients treated with AIs, B. Patients treated with SERDs, C. Patients treated with SERMs, D. Patients treated with EE2. Abbreviations; AI, aromatase inhibitor; SERD, selective estrogen receptor downregulator; SERM, selective estrogen receptor modulator; EE2, ethinyl estradiol.

Longitudinal plasma samples, collected at more than two time-points of the clinical course, from 52 patients were used to look at changes in the presence of *ESR1* and *PIK3CA* mutations during treatment (three points from a total of 12 patients (23.1%), four points from a total of 5 (9.6%), five points from one (1.9%), and six points from one (1.9%) out of 52 MBC patients). The actual changes in the number of copies/μL in *ESR1* mutations and *PIK3CA* mutations in cfDNA during treatment in 52 MBC patients are shown in Supplementary Figure S3 Fourteen patients had *ESR1* mutations and 9 patients had *PIK3CA* mutations in the first blood draw. Over the course of treatment, 10 patients acquired, but 9 patients lost *ESR1* mutations. Meanwhile, 12 patients acquired, but 3 patients lost *PIK3CA* mutations. Thus, there are more *ESR1* and *PIK3CA* mutated patients in the serial analysis than in the snap shot analysis (Supplementary Table S4). Patients were grouped into those who had no *ESR1* or *PIK3CA* mutations over the course of treatment (*N* = 28), those in whom *ESR1* or *PIK3CA* mutations were acquired or maintained (*N* = 15), and those in whom *ESR1* or *PIK3CA* mutations were disappeared after treatment in cfDNA (*N* = 9), and groups were compared by the patient response end-points of duration of ET effectiveness (Figure 3C, 3D). Patients in the loss of cfDNA *ESR1* mutations group had a longer duration of ET effectiveness than patients in the acquired or maintained numbers of cfDNA *ESR1* mutations group, but had a shorter duration of ET effectiveness than patients without mutations over the course of treatment (*P* < 0.0001). On the other hand, there was no statistically significant differences in these three groups; no *PIK3CA* mutations during treatment group (*N* = 31), the loss of cfDNA *PIK3CA* mutations group (*N* = 3), and the acquired or maintained numbers of cfDNA *PIK3CA* mutations group (*N* = 18) (*P* = 0.10). We did not detect any trend towards particular *ESR1* and *PIK3CA* mutation hotspots over the course of treatment, but indicated that polyclonal mutations appeared or disappeared more frequently in *ESR1* than in *PIK3CA* (Supplementary Table S4; appeared polyclonal mutations, 40% for *ESR1* vs 5.6% for *PIK3CA*: disappeared polyclonal mutations, 66.7% for *ESR1* vs 0% for *PIK3CA*). Intrapatient changes of the number of copies/μL of *ESR1* and *PIK3CA* mutations in each representative therapeutic drug group in 21 patients with longitudinal data are shown in Figure 4. Furthers, we listed the treatment just before the latest blood draw as “representative treatment between blood draw” in Table 4. All plasma samples were taken at the time of disease progression, so that it provided an evaluation of *ESR1* and *PIK3CA* mutation status when each ET failed. In patients treated with AIs, 26.7% (4/15) had acquired *ESR1* mutations, which was more frequent compared to the 13.3% (2/15) of patients lost *ESR1* mutations. The two patients acquiring *ESR1* mutations (Pt 33 and Pt 82) developed *PIK3CA* mutations over the course of treatment. In patients treated with selective ER down regulators (SERDs), 62.5% (5/8) showed acquired or maintained numbers of *ESR1* mutations, but 25% (2/8) had decreases in the number of *ESR1* mutations. Of the SERD-treated patients acquiring or maintaining *ESR1* mutations, 60% (3/5) showed increases in *PIK3CA* mutations. In patients treated with SERMs, 50% (2/4) had acquired *ESR1* mutations. In patients treated with ethinyl estradiol (EE2), 40% (2/5) of EE2-treated patients showed decreases in *ESR1* mutations. Interestingly, among ETs, decreases in *PIK3CA* mutations were detected in one SERM-treated patient.

**Table 4:** Comparison between changes of *ESR1* and *PIK3CA* mutations and each therapeutic drug in 52 MBC patients with longitudinal data

Genomic state		No. of patients (%)							
		Representative treatment between blood draws							
		Total	No	AI	SERM	SERD	EE2	MPA	Chemotherapy
		(*N* = 52)	(*N* = 7)	(*N* = 15)	(*N* = 5)	(*N* = 8)	(*N* = 5)	(*N* = 2)	(*N* = 10)
***ESR1***	WT	28 (53.8)	7 (100)	9 (60)	1 (20)	1 (12.5)	2 (40)	1 (50)	7 (70)
	Acquires or maintenance of mutation numbers	15 (28.8)	0	4 (26.7)	2 (40)	5 (62.5)	1 (20)	1 (50)	2 (20)
	Disappears in mutation numbers	9 (17.3)	0	2 (13.3)	2 (40)	2 (25)	2 (40)	0	1 (10)
***PIK3CA***	WT	31 (59.6)	7 (100)	10 (75)	3 (60)	5 (62.5)	2 (40)	1 (50)	3 (30)
	Acquires or maintenance of mutation numbers	18 (34.6)	0	5 (25)	1 (20)	3 (37.5)	3 (60)	1 (50)	5 (50)
	Disappears in mutation numbers	3 (5.8)	0	0	1 (20)	0	0	0	2 (20)

Abbreviations: WT, wild-type; AI, aromatase inhibitor; SERM, selective estrogen receptor modulator; SERD, selective estrogen receptor downregulator; EE2, Ethinyl estradiol; MPA, medroxyprogesterone acetate.

## DISCUSSION

dPCR is a highly sensitive technique for detecting rare mutations, but analysis is on the basis of a single mutation per assay. For screening multiple mutations in a limited amount of sample, an assay to detect multiple mutations in parallel has been demonstrated with dPCR [15, 19, 20]. Here, we used multiplex ddPCR to study a cohort of patients treated with multiple lines of hormonal therapy to determine whether on-treatment *ESR1* and *PIK3CA* mutations can be detected noninvasively and to examine the potential clinical importance of these mutations both in a snapshot and serially.

We verified that up to three mutations in *ESR1* and up to 12 and five mutations in *PIK3CA* could be multiplexed into a single assay, which could detect the three most common mutations in *ESR1* (Y537S, Y537N, and D538G) and 17 mutations in the 5 most common mutation sites of *PIK3CA* (E542K/V, E545V/G/A/Q/K, Q546L/R/P/E/K, H1047L/R/Y, G1049R/S) (Figure 1, Supplementary Figure S4). Toy and colleagues revealed the spectrum of *ESR1* mutations from more than 900 patients and their potential differential impact (e.g. tumors driven by Y537S, but not D538G, E380Q, or S463P, were less effectively inhibited by fulvestrant in comparison with more potent and bioavailable antagonists, including AZD9496 [21]. Therefore, we examined the less frequent *ESR1* mutations, E380Q (LBx^®^ Probe ESR1 E380Q (A081), Riken Genesis, Tokyo, Japan) and Y537C (probe shown in [22]), but we could not detect them in our cohort. In validation assay, some of the mutations are found in the multiplex assay, but not using the uniplex assay (Y537N: 5 detection by multiplex, only 2 by uniplex) (Supplementary Table S1). Because the cut-off level of the presence of the mutation was 3 positive droplets in both multiplex and uniplex assay, this study had the possibility that uniplex assay could not detect a mutation due to less than the cut-off level, which could be detected as one of target mutations using multiplex probe.

The subjects of this retrospective study were a total of 185 plasma samples from 86 ER-positive patients, of which 151 plasma samples were from 69 MBC patients and 34 plasma samples were from 17 advanced PBC patients. Plasma *ESR1* mutations were found in 28.9% (20/69) of MBC patients while plasma *PIK3CA* mutations were found in 24.6% (17/69) of MBC patients, with a distribution of mutations that was highly similar to previously published data [2–4, 19]. Interestingly, only 9 patients had both *ESR1* and *PIK3CA* mutations over the all of treatment. These results were compatible with the recent report that *PIK3CA* was mutated in 37% (53/143) and *ESR1* was mutated in 14% (20/143) of the ER-positive /HER2-negative MBC, in which only 9 had both mutations [23].

All patients with *ESR1* mutations had resistance to prior AI (*P* = 0.0074), and the majority of patients with *ESR1* mutations had resistance to prior SERM therapy (*P* = 0.0087), prior both AI and SERM therapy (*P* = 0.0014), and had received three or more prior endocrine regimens (*P* = 0.041) (Table 2). Detailed information of three or more prior endocrine regimens was shown in Supplementary Table S5. On the other hand, *PIK3CA* mutations in cfDNA were not associated with previous endocrine exposure. Among all samples, 67.4% had polyclonal mutations in *ESR1*, which exhibited markedly more heterogeneity than *PIK3CA* mutations, 11.3% of which were polyclonal (Figure 2A). These findings are compatible with the report that polyclonal *ESR1* mutations were present in 19.2-49.1% of *ESR1* mutant patients [13, 15, 19, 24], but *PIK3CA* mutations were often monoclonal [15, 25].

In this study, we focused on “duration” of ET effectiveness. Because endocrine treatments can be administered repeatedly and consistently in ER-positive MBC patients without life-threating visceral metastases [26], the efficacy of endocrine treatments may contribute to the “duration” of ET effectiveness even if the endocrine treatments during the period varied. Furthers, this analysis had the problem that the time points and the number of samples analyzed were different among the patients. However, we overwhelmed it by comparing *ESR1* mutations with *PIK3CA* mutations because the clinical role of *PIK3CA* mutations becomes clear as follow; *PIK3CA* mutations have the highest frequency in primary and metastatic breast tumors [12] and they are not statistically significant prognostic marker or predictor of ET effectiveness [14, 16]. Patients with detectable plasma *ESR1* mutations (*P* < 0.0001) and *PIK3CA* mutations (*P* = 0.0034) showed significantly shorter duration of ET effectiveness by log-rank test (Figure 3, Supplementary Figure S2). In the Cox hazards model, the presence of *ESR1* mutations in cfDNA was a significant prognostic parameter in both univariate analysis (HR: 3.2, 95% CI: 1.76-5.71, *P* = 0.0002) and in multivariate analysis (HR: 2.04, 95% CI: 1.08-3.83, *P* = 0.029). However, the presence of *PIK3CA* mutations in cfDNA was a significant prognostic parameter in univariate analysis only (Table 3).

Potential interest of monitoring *ESR1* mutations in the metastatic setting has been increasing. Recently, Clatot and colleagues reported that cfDNA *ESR1* mutations are independent risk factors for poor outcome after AI failure, and they are frequently detectable before clinical progression [27]. On the other hand, Spoerkle and colleagues did not show clinical utility of *ESR1* mutations as a monitoring tool [24]. In our tracking cfDNA *ESR1* mutations and *PIK3CA* mutations study, patients in whom the number of cfDNA *ESR1* mutations were lost had a longer duration of ET effectiveness than patients in whom the numbers of cfDNA *ESR1* mutations were acquired or maintained, but had a shorter duration of ET effectiveness than patients without mutations over the course of treatment (*P* < 0.0001). On the other hand, there was no statistically significant differences in these three groups; no *PIK3CA* mutations during treatment group (*N* = 31), the loss of cfDNA *PIK3CA* mutations group (*N* = 3), and the acquired or maintained numbers of cfDNA *PIK3CA* mutations group (*N* = 18) (*P* = 0.10). These differences regarding the prevalence of *ESR1* and *PIK3CA* mutations may be caused by polyclonal breast tumor evolution under the selective pressure of ET [24]. *ESR1* mutations occur late in endocrine treatment and in a subclonal manner, so that these mutations are generally detected in metastatic lesions [2–4, 22]. In contrast, most *PIK3CA* mutations occur early in the process of tumor development and its status does not change in the majority of patients who develop recurrent or progressive breast cancer [28] (Figure 4).

The present study has limitations. This was a retrospective, single-institute study, and was prone to selection bias. The studied population was heterogeneously treated and all plasma samples were taken at the time of disease progression, so that we had insufficient data to examine whether or not *ESR1* mutation detection is dependent on specific hormone therapies. The samples used in this study were obtained for biobanking. Therefore, the time from blood draw to spinning, freezing plasma and then thawing may affect the variability of the data.

In conclusion, our study demonstrates the clinical significance of the burden of on-treatment hotspot *ESR1* LBD mutations, both in a snapshot and serially in MBC patients in comparison with *PIK3CA* hotspot mutation status, using multiplex ddPCR assays.

## MATERIALS AND METHODS

### Patients and breast cancer samples

A total of 86 patients (185 plasma samples) with breast carcinoma, treated at Kumamoto University Hospital between 2003 and 2016, were enrolled in this study. Cases were selected if archival plasma samples were available. Informed consent was obtained from all patients before biopsy or surgery. The Ethics Committee of Kumamoto University Graduate School of Medicine (Kumamoto, Japan) approved the study protocol. Adjuvant and neoadjuvant treatment was administered in accordance with the recommendations of the St. Gallen international expert consensus on the primary therapy of early BC [29–31]. The treatment of MBC patients was performed in accordance with the National Comprehensive Cancer Network Clinical Practice Guidelines in Oncology [26]. Recurrence was defined as the identification of positive spots by physical examination and/or by imaging diagnosis during the follow-up period. Patients were examined at the Kumamoto University Hospital or affiliated hospitals every 3 months for 5 years and every year thereafter and they were assessed monthly or at longer intervals depending on their disease status.

### Sample preparation

Blood collected in EDTA K_2_ tubes was processed as soon as possible and was centrifuged at 1,467 g for 10 min, with plasma stored frozen until DNA extraction. DNA was extracted from 500 μL aliquots of plasma using the ISOSPIN Blood & Plasma DNA kit (Nippon Gene, Tokyo, Japan) according to the manufacturer's instructions. All DNA extracts were quantified using a NanoDrop 2000 spectrometer (NanoDrop Technologies, Wilmington, DE, USA) and purity was determined from the A260/A280 absorbance ratios.

### Analysis of ESR1 mutations by ddPCR

We performed duplicate ddPCR assay on a QX200 digital PCR system (Bio-Rad laboratories, Hercules, CA, USA) using the assays as described previously [17, 32]. The PCR data were quantified as copies/μL and fractional abundance (allele frequency) using QuantaSoft^™^ software (Bio-Rad laboratories). A mutation was considered positive with more than three *ESR1* mutant or *PIK3CA* droplets. The uniplex ddPCR method had been optimized beforehand by comparative analysis of a dilution series of synthetic copies of each indicated mutant *ESR1* oligonucleotide, as reported previously [17, 22].

### Probes and primers

We used LBx^®^ Probe ESR1 Multi (A082) as the detection probe for *ESR1* Y537S/Y537N, and D538G, LBx^®^ Probe PIK3CA Screen1 (A087) as the detection probe for *PIK3CA* E542K/V and E545V/G/A/Q/K, Q546L/R/P/E/K, and LBx^®^ Probe PIK3CA Screen2 (A088) as the detection probe for *PIK3CA* H1047L/R/Y and G1049R/S (Riken Genesis, Tokyo, Japan), and Custom *Taq*Man SNP Genotyping assays (Applied Biosystems, Foster City, CA, USA) for the detection of other *ESR1* LBD mutations (Y537S, Y537N, and D538G), as described previously [22].

### Immunohistochemistry

Immunohistochemical staining was carried out on 4-μm thick tumor sections. Serial sections were prepared from selected blocks and float-mounted on adhesive-coated glass slides for ERα, PgR, HER2, and Ki67 staining. Primary antibodies, their visualization methods, and their evaluation were as previously described [33].

### Statistical analysis

The chi-square test or Fisher's exact test was used to assess baseline differences between binary variables. Correlations were calculated using Spearman's rank correlation coefficient. In the analysis of duration of ET effectiveness, the Kaplan-Meier method was used to estimate survival rates, and differences between survival curves were evaluated by the log-rank test. Cox's proportional hazards model was used for the univariate and multivariate analysis of prognostic status. *P* values < 0.05 were considered a significant result. All reported *P* values are two-sided, and CIs are at the 95% level. All statistical analyses were two-sided and performed using JMP software version 10.0.1 for Windows (SAS institute Japan, Tokyo, Japan).

## SUPPLEMENTARY MATERIALS FIGURES AND TABLES












