# Supplementary material for: Analysis of ESR1 and PIK3CA mutations in plasma cell-free DNA from ER-positive breast cancer patients
Source: Oncotarget. 2017 Jun 14;8(32):52142–55. doi: 10.18632/oncotarget.18479 (PMC5581019; doi:10.18632/oncotarget.18479)
Supplement: Supplementary file 1 [file oncotarget-08-52142-s001.pdf]

Analysis of *ESR1* and *PIK3CA* mutations in plasma cell-free DNA from ER-positive breast cancer patients

Supplementary Material

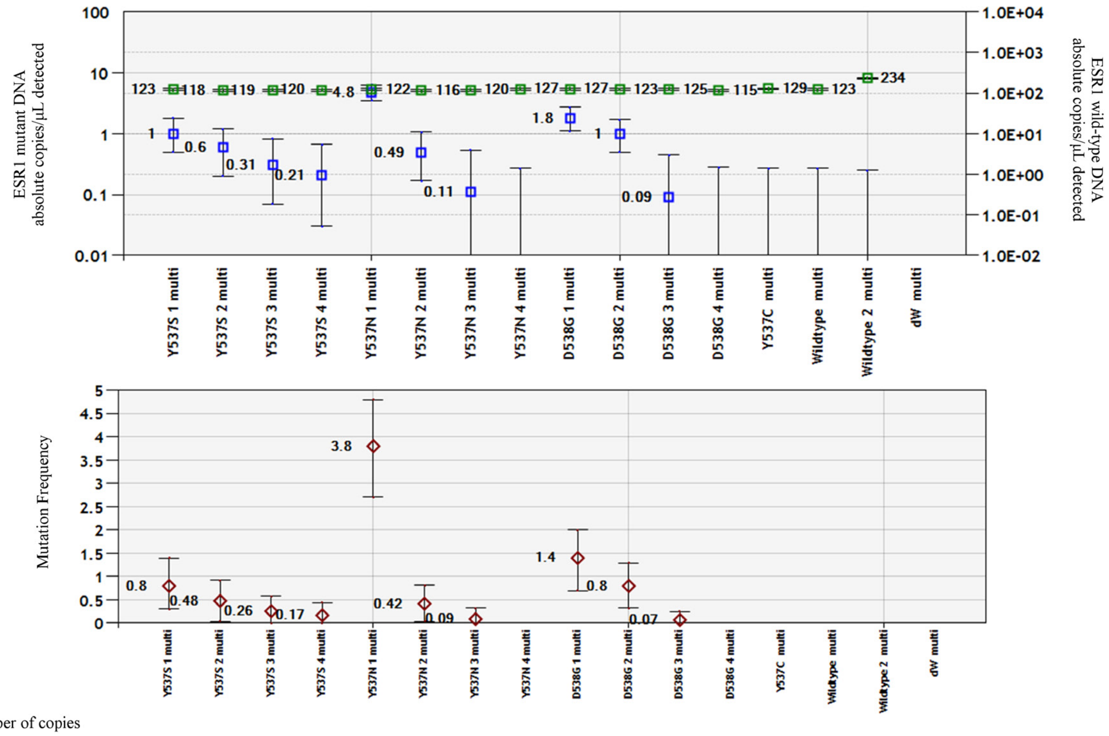

Number of copies																
Mutant DNA	10	5	3	2	46	5	1	0	18	11	1	0	0	0	0	0
ESR1 wild-type DNA	1112	985	1086	1097	1120	1118	1004	1359	1230	1289	1277	1170	1335	1293	2517	0

Supplementary Figure 1: Comparative analysis of the dilution series and cross-reactivity of each indicated synthetic *ESR1* mutation oligonucleotide in a background of wild-type normal human DNA using the multiplex detection probe. The upper panel shows the absolute number of copies/μL of each indicated synthetic *ESR1* mutation oligonucleotide and wild-type normal human DNA and the lower panel shows corresponding fractional abundance (%). The dilution experiments were prepared by two-fold serial dilution of each synthetic *ESR1* mutation stock oligonucleotide in a background of wild-type normal human DNA (TaqMan Control Genomic DNA) where the total DNA content of each ddPCR reaction was 20 ng and “wild-type 2” was 40 ng. There was no cross-reactivity for the detection of any of the *ESR1* mutations. We confirmed that this assay was able to detect each *ESR1* mutant molecule in a background of wild-type normal human DNA at the lowest concentration and was not able to detect any false-positives in the wild-type normal human DNA. Abbreviation; dW, distilled water.

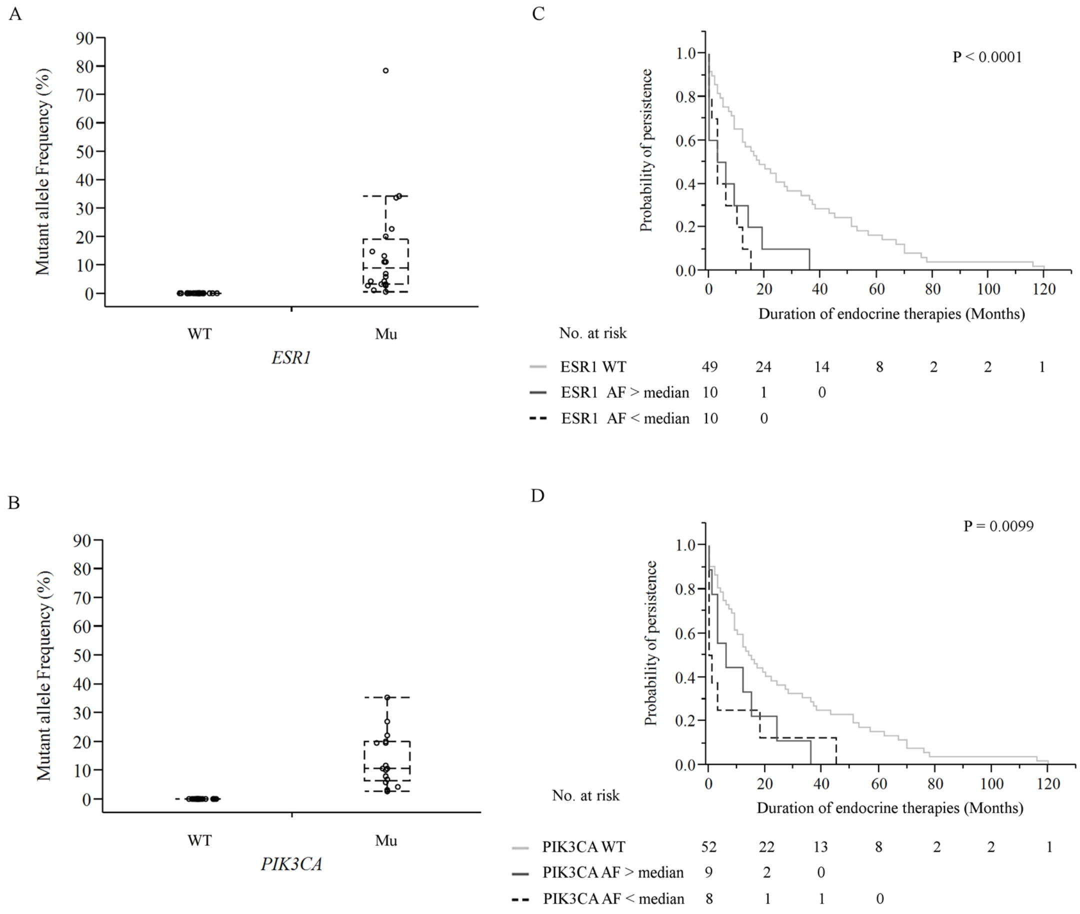

Supplementary Figure 2: A, B. Box-plots of the AF of *ESR1* mutations and *PIK3CA* mutations. The median AF of *ESR1* mutations was 8.66% (range, 0.35–78.5) (A) and the median AF of *PIK3CA* mutations was 10.3% (range, 2.57–35) (B). C, D. Kaplan-Meier plots of the association of the AF of *ESR1* mutations (C) and *PIK3CA* mutations (D) in cfDNA with duration of ET effectiveness in this cohort. The dichotomized *ESR1* and *PIK3CA* AFs (cutoff each AF > median) did not show a clear difference in duration of ET effectiveness. Abbreviations; AF, allele frequency; cfDNA, cell-free DNA; WT, wild-type; Mu, mutation; ET, endocrine therapy.

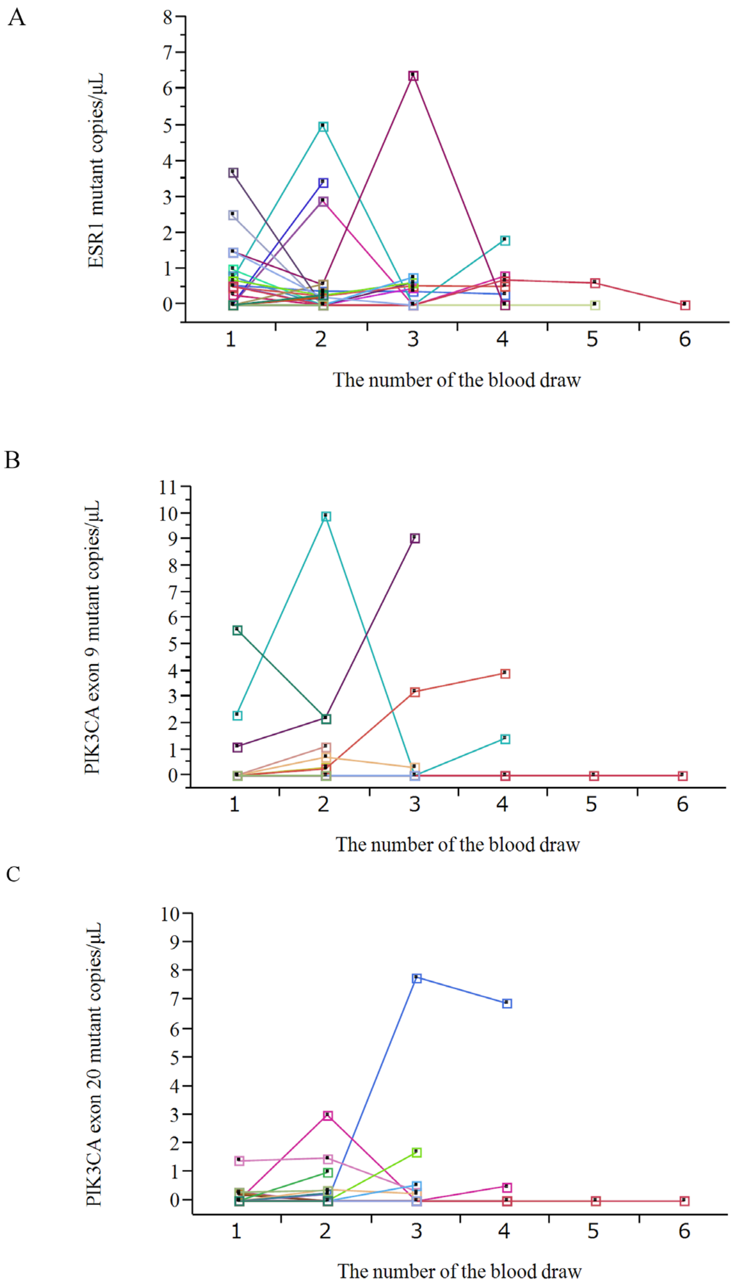

Supplementary Figure 3: A-C. show the actual changes in the number of copies/µL in *ESR1* mutations and *PIK3CA* mutations in cfDNA during treatment in 52 MBC patients. (A: *ESR1* Y537S, Y537N, and D538G, B: *PIK3CA* E542K/V and E545V/G/A/Q/K, Q546L/R/P/E/K, C: *PIK3CA* H1047L/R/Y and G1049R/S). Abbreviation; cfDNA, cell-free DNA.

A

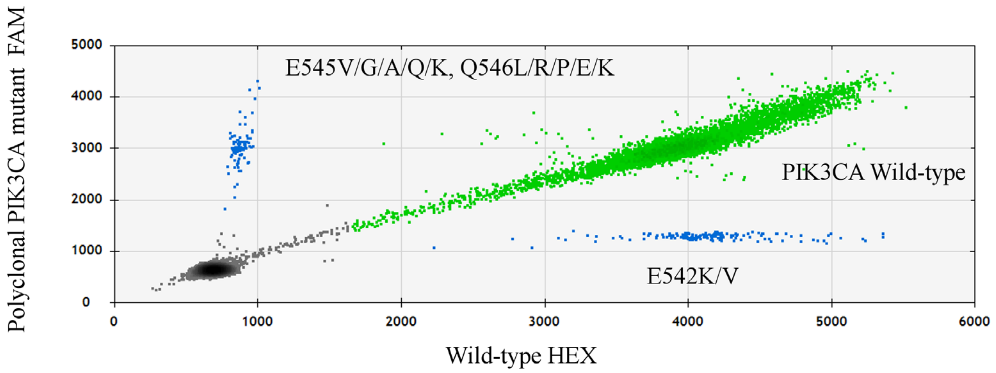

B

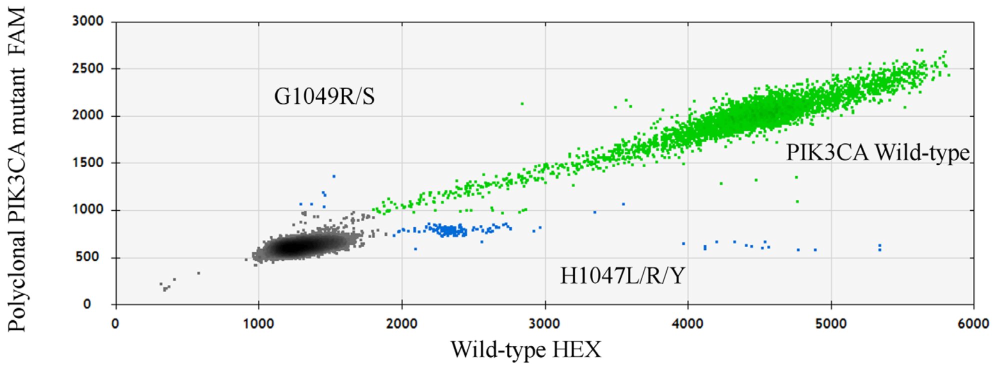

Supplementary Figure 4: A, B. Representative ddPCR analysis of polyclonal *PIK3CA* mutations (A: E542K/V and E545V/G/A/Q/K, Q546L/R/P/E/K, B: H1047L/R/Y and G1049R/S). In each plot, green dots represent HEX-labeled wild type DNA, blue dots represent FAM-labeled mutant DNA, and black dots are droplets with no DNA incorporated.

For Supplementary Tables see in Supplementary Files.
