# Supplementary material for: Analysis of ESR1 and PIK3CA mutations in plasma cell-free DNA from ER-positive breast cancer patients
Source: Oncotarget. 2017 Jun 14;8(32):52142–55. doi: 10.18632/oncotarget.18479 (PMC5581019; doi:10.18632/oncotarget.18479)
Supplement: Supplementary file 2 [file oncotarget-08-52142-s002.docx]

Table S1 Comparison of single and polyclonal LBD *ESR1* mutations between uniplex and multiplex ddPCR assays from a validation subset of 26 women (62 blood samples).

|  | No. of samples (%) | | |
| --- | --- | --- | --- |
| ***ESR1* genomic state** | *ESR1* mutant detection probe | | |
|  | Multiplex | Uniplex | Kappa value |
|  | (*N* = 13 ) | (*N* = 11 ) |  |
| Y537S | 3 (23.1) | 3 (27.3) | 1.0 |
| Y537N | 5 (38.5) | 2 (18.2) | 0.55 |
| D538G | 9 (69.2) | 6 (54.5) | 0.77 |
| **Polyclonal mutation** |  |  |  |
| Y537S/Y537N | All detected | Y537S only |  |
| Y537S/Y537N/D538G | All detected | Y537S only |  |
| Y537N/D538G | All detected | Not detected |  |

Abbreviations: LBD, ligand binding domain; ddPCR, droplet digital polymerase chain reaction.
