# Supplementary material for: Analysis of ESR1 and PIK3CA mutations in plasma cell-free DNA from ER-positive breast cancer patients
Source: Oncotarget. 2017 Jun 14;8(32):52142–55. doi: 10.18632/oncotarget.18479 (PMC5581019; doi:10.18632/oncotarget.18479)
Supplement: Supplementary file 3 [file oncotarget-08-52142-s003.docx]

|  |  | **No. of samples (%)** | | | | | | |
| --- | --- | --- | --- | --- | --- | --- | --- | --- |
|  |  | ***ESR1* genomic state** | | | | | | |
|  | Total | WT | **Monoclonal mutation** | | | **Polyclonal mutation** | | |
|  |  |  | D538G | Y537S | Y537N | D538G/Y537S  /Y537N | Y537S/Y537N | D538G/Y537N |
| ***PIK3CA* genomic state** | (*N* = 185) | (*N* = 139) | (*N* = 7) | (*N* = 5) | (*N* = 3) | (*N* = 23) | (*N* = 7) | (*N* = 1) |
| WT | 141 (76.2) | 110 (79.1) | 2 (28.6) | 3 (60) | 3 (100) | 17 (73.9) | 6 (85.7) | 0 |
| **Monoclonal mutation** | | | | | | | | |
| H1047x | 26 (14.1) | 14 (14.4) | 2 (28.6) | 0 | 0 | 3 (13) | 0 | 1 (100) |
| E545x, Q546x | 7 (3.8) | 2 (1.4) | 2 (28.6) | 1 (20) | 0 | 2 (8.7) | 0 | 0 |
| E542x | 5 (2.7) | 3 (2.2) | 0 | 0 | 0 | 1 (4.6) | 1 (14.3) | 0 |
| G1049x | 1 (0.54) | 0 | 0 | 1 (20) | 0 | 0 | 0 | 0 |
| **Polyclonal mutation** | | | | | | | | |
| E542x/E545x, Q546x | 2 (1.1) | 0 | 1 (14.3) | 0 | 0 | 0 | 0 | 0 |
| E542x/ E545x, Q546x/H1047x | 1 (0.54) | 1 (0.72) | 0 | 0 | 0 | 0 | 0 | 0 |
| E542x/H1047x | 1 (0.54) | 1 (0.72) | 0 | 0 | 0 | 0 | 0 | 0 |
| H1047x/G1049x | 1 (0.54) | 0 | 0 | 0 | 0 | 0 | 0 | 0 |

Table S2. Comparison of the number of copies/μL between *ESR1* mutations and *PIK3CA* mutations in plasma cfDNA.

Abbreviations: cfDNA, cell-free DNA; WT, wild-type; H1047x, H1047L/R/Y; E545x Q546x, E545V/G/A/Q/K Q546L/R/P/E/K; E542x, E542K/V; G1049x, G1049R/S.
