# Supplementary material for: Analysis of ESR1 and PIK3CA mutations in plasma cell-free DNA from ER-positive breast cancer patients
Source: Oncotarget. 2017 Jun 14;8(32):52142–55. doi: 10.18632/oncotarget.18479 (PMC5581019; doi:10.18632/oncotarget.18479)
Supplement: Supplementary file 4 [file oncotarget-08-52142-s004.docx]

Table S3. Patient characteristics associated with *PIK3CA* mutations in PBC.

|  | No. of patients (%) | | | |
| --- | --- | --- | --- | --- |
| **Variables** | Total | *PIK3CA* | |  |
|  |  | Wild-type | Mutant^$^ | *P*-value |
|  | (*N* = 17 ) | (*N* = 13 ) | (*N* = 4 ) |  |
| **Age at biopsy** |  |  |  |  |
| Median (range) | 67 (41–82) | 71 (62.5–75.5) | 62.5 (45.5–78) | 0.49 |
| **Clinical Stage at diagnosis** |  |  |  |  |
| I | 4 (23.5) | 4 (30.7) | 0 | 0.35 |
| II | 11 (64.7) | 8 (61.5) | 3 (75) |  |
| III | 2 (11.8) | 1 (7.7) | 1 (25) |  |
| **Histological type** |  |  |  |  |
| Invasive ductal | 13 (76.5) | 11 (84.6) | 2 (50) | 0.14 |
| Invasive lobular | 1 (5.9) | 0 | 1 (25) |  |
| Mucinous | 3 (17.6) | 2 (15.4) | 1 (25) |  |
| **Histological grade** |  |  |  |  |
| 1 | 5 (29.4) | 3 (23.1) | 2 (50) | 0.071 |
| 2 | 11 (64.7) | 10 (76.9) | 1 (25) |  |
| 3 | 0 | 0 | 0 |  |
| Lobular | 1 (5.9) | 0 | 1 (25) |  |
| **Median percentage of ERα median (25%, 75%)** | 90 (90–95) | 90 (85–95) | 92.5 (90–98.8) | 0.40 |
| **Median percentage of PgR median (25%, 75%)** | 50 (7.5–80) | 50 (10–80) | 25 (0–87.5) | 0.49 |
| **HER2** |  |  |  |  |
| Negative | 16 (94.1) | 12 (92.3) | 4 (100) | 0.57 |
| Positive | 1 (5.9) | 1 (7.7) | 0 |  |
| **Prior endocrine therapy** |  |  |  |  |
| AI | 17 (100) | 13 (100) | 4 (100) |  |

^$^ *PIK3CA* mutations were 3 H1047L/R/Y and 1 H1047 L/R/Y and G1049R/S.

Abbreviations: PBC, primary breast cancer; ERα, estrogen receptor alpha; PgR, progesterone receptor; HER2, human epidermal growth factor receptor 2; AI, aromatase inhibitor.
