# Supplementary material for: Analysis of ESR1 and PIK3CA mutations in plasma cell-free DNA from ER-positive breast cancer patients
Source: Oncotarget. 2017 Jun 14;8(32):52142–55. doi: 10.18632/oncotarget.18479 (PMC5581019; doi:10.18632/oncotarget.18479)
Supplement: Supplementary file 5 [file oncotarget-08-52142-s005.docx]

Table S4. Details of the change of *ESR1* and *PIK3CA* mutations in 52 metastatic breast cancer patients with longitudinal samples.

| No. of samples (%) | | | |
| --- | --- | --- | --- |
| **Increase/maintenance of the number of mutations** |  | **Decrease in the number of mutations** |  |
| ***ESR1*** | (*N* = 15 ) | ***ESR1*** | (*N* = 9 ) |
| D538G→D538G | 2 (13.3) | Y537S→WT | 1 (11.1) |
| Y537S/Y537N/D538G→Y537S | 1 (6.7) | Y537N→WT | 1 (11.1) |
| Y537S/Y537N/D538G→Y537S/Y537N/D538G | 2 (13.3) | D538G→WT | 1 (11.1) |
| WT→Y537S | 2 (13.3) | Y537S/Y537N→WT | 2 (22.2) |
| WT→Y537N | 1 (6.7) | Y537S/Y537N/D538G→WT | 4 (44.4) |
| WT→D538G | 1 (6.7) |  |  |
| WT→Y537S/Y537N | 2 (13.3) |  |  |
| WT→Y537S/Y537N/D538G | 4 (26.7) |  |  |
| ***PIK3CA*** | (*N* = 18 ) | ***PIK3CA*** | (*N* = 3 ) |
| E542x→E542x | 2 (11.1) | H1047x→WT | 3 (100) |
| E545x, Q546x→E545x, Q546x | 1 (5.6) |  |  |
| H1047x→H1047x | 3 (16.7) |  |  |
| WT→E545x, Q546x | 3 (16.7) |  |  |
| WT→H1047x | 7 (38.9) |  |  |
| WT→G1049x | 1 (5.6) |  |  |
| WT→E542x/ E545x, Q546x/H1047x | 1 (5.6) |  |  |

Abbreviations: WT, wild-type; E542x, E542K/V; E545x Q546x, E545V/G/A/Q/K Q546L/R/P/E/K; H1047x, H1047L/R/Y; G1049x, G1049R/S.
