# Supplementary material for: Analysis of ESR1 and PIK3CA mutations in plasma cell-free DNA from ER-positive breast cancer patients
Source: Oncotarget. 2017 Jun 14;8(32):52142–55. doi: 10.18632/oncotarget.18479 (PMC5581019; doi:10.18632/oncotarget.18479)
Supplement: Supplementary file 6 [file oncotarget-08-52142-s006.docx]

Table S5. The detailed information in the patients with *ESR1* or *PIK3CA* mutations received 3 or more endocrine treatments prior to the first blood draw.

| Case No. |  | *ESR1* genomic status | *PIK3CA* genomic status |
| --- | --- | --- | --- |
| 2 | TAM→EXE→LET→EE2 | Wild-Type | E545x Q546x |
| 4 | ANA→EXE→MPA→LET→SERD | Y537S/N, D538G | Wild-Type |
| 5 | TAM→FAD→ANA→EXE→TAM→LET→ANA→HdTOR→LET→SERD | Wild-Type | H1047x |
| 6 | LET→HdTOR→ANA→MPA | Y537S/N | H1047x |
| 13 | LHRHa+TAM→ANA→EXE→HdTOR→MPA→ANA→EE2 | Y537S/N | E542x |
| 14 | TAM→LHRHa+TAM→LET | Y537S/N | Wild-Type |
| 15 | LET→HdTOR→SERD | Y537S/N, D538G | H1047x |
| 21 | LET→HdTOR→EXE→SERD | Y537S/N, D538G | Wild-Type |
| 25 | TOR→EXE→TOR | Y537S/N, D538G | Wild-Type |
| 31 | LET→HdTOR→ANA→SERD | Y537S/N, D538G | H1047x |
| 32 | ANA→HdTOR→LET→SERD | Y537S/N, D538G | Wild-Type |
| 33 | LET→SERD→HdTOR→EXE→ANA | Y537S/N, D538G | E542x |
| 36 | LET→EXE→LET→HdTOR→ANA→SERD | Y537S/N, D538G | Wild-Type |
| 38 | LHRHs+TAM→LHRHa+ANA→SERD | Y537S/N, D538G | E545x Q546x |
| 67 | TAM→LET→HdTOR→ANA | Wild-Type | E545x Q546x |
| 68 | AI*→TAM→LET | D538G | E545x Q546x |
| 72 | ANA→EXE→HdTOR→LET | Wild-Type | H1047x |
| 74 | EXE→SERD→LHRH+ANA→HdTOR→MPA→LET→HdTOR | Y537N | Wild-Type |
| 76 | TOR→ANA→EXE→MPA→TAM→LET→ANA→HdTOR→EE2 | Wild-Type | H1047x |
| 82 | TAM→LHRHa+EXE→LHRHa+HdTOR→LHRHa+LET→MPA→ANA | D538G | H1047x |
| 86 | TAM→LET→EXE | D538G | Wild-Type |

* Details are unknown

Abbreviations; E545x Q546x, E545V/G/A/Q/K Q546L/R/P/E/K; E542x, H1047x, H1047L/R/Y; E542K/V; TAM, tamoxifene; EXE, exemestane; LET, letrozole; EE2, ethinyl estradiol; ANA, anastrozole; MPA, Medroxyprogesterone acetate; LET, letrozole; SERD, selective estrogen receptor down regulator; FAD, fadrozole; HdTOR, high dose toremifene; LHRHa,  luteinizing Hormone-Releasing Hormone Analog.
